# ABCG2 in Acute Myeloid Leukemia: Old and New Perspectives

**DOI:** 10.3390/ijms24087147

**Published:** 2023-04-12

**Authors:** Daniela Damiani, Mario Tiribelli

**Affiliations:** 1 Division of Hematology and Stem Cell Transplantation, Udine Hospital, P.le Santa Maria della Misericordia, 5, 33100 Udine, Italy; 2 Department of Medicine (DAME), University of Udine, 33100 Udine, Italy

**Keywords:** acute myeloid leukemia, multidrug resistance, ABCG2, prognosis, survival, counteraction

## Abstract

Despite recent advances, prognosis of acute myeloid leukemia (AML) remains unsatisfactory due to poor response to therapy or relapse. Among causes of resistance, over-expression of multidrug resistance (MDR) proteins represents a pivotal mechanism. ABCG2 is an efflux transporter responsible for inducing MDR in leukemic cells; through its ability to extrude many antineoplastic drugs, it leads to AML resistance and/or relapse, even if conflicting data have been reported to date. Moreover, ABCG2 may be co-expressed with other MDR-related proteins and is finely regulated by epigenetic mechanisms. Here, we review the main issues regarding ABCG2 activity and regulation in the AML clinical scenario, focusing on its expression and the role of polymorphisms, as well as on the potential ways to inhibit its function to counteract drug resistance to, eventually, improve outcomes in AML patients.

## 1. Introduction

Acute myeloid leukemia (AML) is an aggressive heterogenous disease arising from the accumulation and clonal expansion of somatic-driven mutations in CD34+/CD38- hematopoietic progenitors, which demonstrate increased proliferation, survival, and impaired maturation capacity [1,2]. It is the most common form of acute leukemia in adults, with a median age at diagnosis of 68 years and a sharp increase in incidence in the following decades [3]. In addition, AML prevalence is increased by therapy-related AML, which accounts for 10–15% of newly diagnosed AML, an effect of improved survival after anti-cancer therapies [4]. The estimated 5-year overall survival (OS) of AML is around 30%, with great differences between age groups (≈50% in younger patients and only ≈10% in elderly patients) and with disappointing progress over the past five decades, particularly in the older population [5,6]. In recent years, however, a large amount of information has been acquired on the molecular landscape of AML, on its morphologic and immunophenotypic heterogeneity, and on inherited germline predisposition. These data led to a refinement of genetic risk classification and highlight the prognostic importance of initial response to chemotherapy and of the persistence of minimal residual disease [7]. Moreover, moving from the newly identified molecular alterations, various target drugs have been recently developed and approved, and preliminary but increasing evidence suggests the potential for a significant effect on AML outcomes [7,8].

Despite this, the occurrence of intrinsic or acquired drug resistance still results in AML being an aggressive disease and a major challenge for clinicians. Clinical multidrug resistance (MDR) is a multi-factorial phenomenon depending on host variations, drug–drug interaction, deregulation of cell death mechanisms, failure of DNA damage response and repair, epigenetic alterations, intratumor heterogeneity, and microenvironment alteration. MDR protects leukemia cell from immune surveillance and from the alteration in intracellular drug concentrations due to overexpression of membrane drug transporter proteins [9]. Acquired MDR has been intensively studied, and the molecular basis of this phenomenon is well established [10]. The cross-resistance to different, structurally unrelated anti-cancer drugs, such as vinblastine, vincristine, and daunomycin, developed by Chinese hamster lung cells grown in actinomycin D to select resistant cells, was first described more than 50 years ago [11]. A few years later, another study reported that daunomycin was actively transported out of multidrug-resistant cells [12]. The hypothesis of a promiscuous membrane transporter able to confer MDR was confirmed by the identification in Chinese hamster ovary cells of the “P-glycoprotein”, so called for the altered membrane permeability associated with its expression in resistant cells [13] and by characterization of its encoding gene [14]. The human homologue gene was soon identified and referred to as ATP-binding cassette (ABC) subfamily B1, ABCB1 [15]. Its recognition paved the way to studies of ABC transporters, leading to the identification of 48 human membrane proteins, grouped into 7 subfamilies, involved in different physiological biochemical and developmental processes beyond cancer drug transport [16,17]. The ABC superfamily is highly conserved among plant and animal species, mainly acting as import pumps in prokaryotes [18]. In eukaryotic cells, they act as exporters, pumping out substances from the cytoplasm or entrapping them into intracellular organelles, such as peroxisome, endoplasmic reticulum, or lysosomes, using an energy-dependent process involving binding and hydrolysis of ATP [19,20].

**Figure 1:**
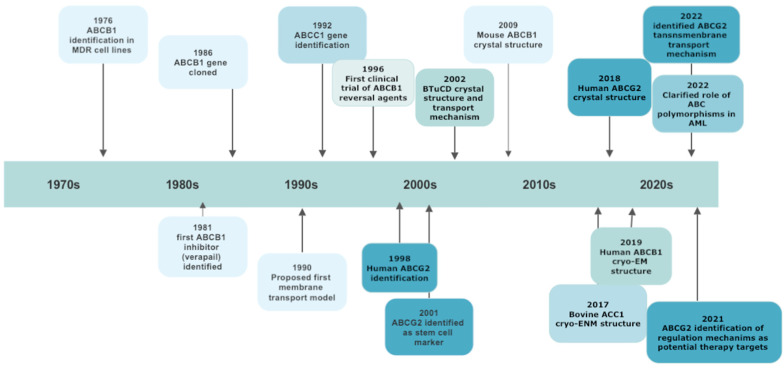
A schematic chronology of significant discoveries of ABC transporters involved in multidrug resistance.

The general architecture of ABC proteins consists of two cytoplasmic nucleotide-binding domains (NBDs), which bind and hydrolyze ATP, and of two sets of hydrophobic transmembrane domains (TMDs), which transport substrates. ABC genes encode either a full transporter or a half transporter with a single TMD domain and a single NBD. Half transporters must dimerize as either homo- or heterodimers to form an active protein [17]. Whereas the structure and function of NBDs are similar throughout ABC subfamilies, TMDs are highly heterogeneous, thus permitting the recognition of different substrates and their translocation across membranes, irrespective of concentration gradient [21]. Despite the huge amount of data on ABC structure obtained by electron microscopy, the precise translocation mechanism remains elusive. It should be underlined that among the 48 ABC members, some have “narrow” substrate specificity, while others (19 of the 48) have broad specificity and are able to transport a wide range of anticancer drugs and to cause drug resistance if overexpressed in tumor cells [22]. The study of “null” mutants established via germline mutations has underscored the diversity of their physiological role and the consequences of their dysfunction. So, at present, more than 20 ABC proteins, belonging to all the identified sub-families, have been associated with human diseases [17,19]. The most important steps in ABC family discoveries are reported in Figure 1.

The present paper will focus on the role of one specific MDR protein, ABCG2, in AML, summarizing the current knowledge on the impact of its overexpression on disease outcome and on the possibility to counteract its action to improve the efficacy of anti-leukemic therapies.

## 2. ABCG Subfamily

The ABCG subfamily includes five ABC half transporters: ABCG1, ABCG2, ABCG4, ABCG5, and ABCG8.

### 2.1. ABCG1

*ABCG1* is coded on chromosome 21q.22.3 and contributes to cholesterol transport and to cellular cholesterol homeostasis [23]. The protein is expressed in many cell types, including endothelial cells, lymphocytes, and myeloid cells on the cell membrane and in endosomes [24]. ACBG1 seems to regulate T cell development in the thymus by regulating intracellular cholesterol levels [25,26]. Moreover, ABCG1 is involved in innate immune response by the regulation of inflammation via reduction of inflammatory cytokines, and in anti-tumor immunity by favoring IL-4–mediated macrophage M2 polarization, producing a pro-cancer effect [27]. Moreover, it seems to be upregulated in lung cancer tissue, and aberrant expression of ABCG1 in lung cancer cells promotes proliferation, migration, and tumor invasion [28]. Roundhill et al., demonstrated ABCG1 expression in osteosarcoma cancer stem cells, suggesting that targeting ABCG1 could improve clinical outcomes [29]. Pan et al., found ABCG1 expression in triple-negative breast cancer, suggesting that it could be used as biomarker in this subset [30]. No data are available at present on the expression of ABCG1 in leukemic cells.

### 2.2. ABCG4

*ABCG4* is mainly localized in the central nervous system (CNS) and seems to have a protective role in Alzheimer’s disease through inhibitory effects on amyloid β production [31]. Furthermore, ABCG4 overexpression seems to confer drug resistance, despite the mechanism not being completely understood [32].

### 2.3. ABCG5 and ABCG8

*ABCG5* and *ABCG8* form an obligated heterodimeric complex (G5/G8), highly expressed on epithelial cells of the intestine and liver, where it mediates sterol transport [33]. Gene variations are associated with hypercholesterolemia, platelet dysfunction, sitosterolemia, cardiovascular disease, and gallstones [34,35,36].

### 2.4. ABCG2

ABCG2 is the most studied among ABCG members. In the early 1990s, the observation of MDR in cell lines selected with mitoxantrone lacking MDR1 and MRP1 expression led to identification of a new transporter protein. The gene responsible for the novel resistance phenotype, first cloned by Doyle et al., in the MCF-7 Adr/Vp cell line, was named *BCRP* for breast cancer resistance protein, from the cell line origin [37,38,39], and further designed as *ABCG2* by the Human Genome Organization Committee. The *ABCG2* gene is highly conserved among species, most of which have a single gene present [40]. The exceptions are rodent and fish, which have more *ABCG2* genes [41]. The human ABCG2 gene is located on chromosome 4, band 4q21–4q22, and extend over 66 kb containing 16 exons (range size from 60 to 532 bp) and 15 introns. The translational start site is on exon 2, ABC signature motif in exon 6, and the ATP binding sites (Walker motif A and B) in exon 3 and exon 6. The promoter region is located approximately 312 bp from the transcriptional start site [42]. The molecular mechanisms controlling *ABCG2* expression are not completely understood, but cell lines with high *ABCG2* expression harbor multiple gene rearrangements in chromosome 4, including gene amplification and translocations [43]. Moreover, there could be a transcriptional regulation, supported by the presence of *cis* regulatory elements in the promoter regions, including an estrogen response element (ERE), a progesterone response element (PRE), a hypoxia response element (HRE), an antioxidant response element (ARE), an aryl carbon response element (AhRE), and the active nuclear factor kB (NFkB) response element [44].

**Figure 2:**
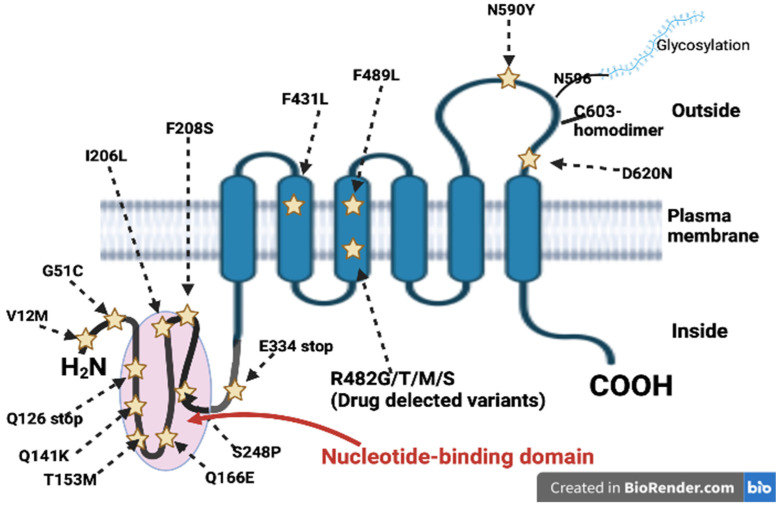
Schematic representation of ABCG2 proteins and its variants. Stars identify single-nucleotide polymorphisms. Abbreviation: NBD: Nucleotide-binding domain.

In vitro studies demonstrated the upregulation of the *ABCG2* gene under hypoxic conditions by estradiol progesterone and by aryl hydrocarbon receptor agonists [45,46,47,48]. *ABCG2* expression can also be induced via peroxisome proliferator-activated receptor γ (PPARγ) [49] and downregulated by dexamethasone via the glucocorticoid receptor (GR) [50]. However, data on *ABCG2* regulation are often controversial, and it has been hypothesized that the observed contradiction may be due to cell- or organ-specific regulation. Epigenetic regulation has been observed in overexpressing cell lines, where elevated *ABCG2* levels were associated with hypomethylation or unmethylation of the CpG island and with hyperacetylation of the *ABCG2* promoter [51]. Furthermore, several microRNAs, such as miR519c, miR520h, and miR328 affect transcription stability and protein translation [52,53,54,55]. Once translated, ABCG2 must multimerize and translocate to the cell membrane to exert its efflux pump function. PI3K/AKT and NFkB pathways and Pim-1 phosphorylation are involved into surface transfer of the protein [56,57]. In the cell membrane, the ABCG2 protein is in a reverse configuration compared to most other ABC transporters, as the ATP binding domain is at the N-terminus and the six putative transmembrane domains are at the C-terminus [58] (Figure 2).

At least one dimerization is required to produce a functional protein; dimerization is under the control of the endoplasmic reticulum quality control (ERQC) network. If passing the ERQC, protein traffics to the Golgi apparatus, where the protein becomes fully glycosylated, passes a further quality control, and can be delivered to its destination, the plasma membrane. Mechanisms by which the protein is transferred to the cell membrane are not completely elucidated and may include direct delivery, trafficking via the endosomal pool or trans-cytotic pathway via the basolateral membrane. Surplus protein is proteolytically degraded in lysosomes, and misfolded proteins become ubiquitinated and degraded in proteosome. Interestingly, 40–60% of the produced protein, even in wild form, does not pass ERQC and is eliminated [59]. Differently to ABCG5/ABCG8, ABCG2 may work only as homodimer; however, several studies have reported that it can assemble in higher oligomeric forms, from tetramers to dodecamers, a process sometimes favored by the presence of single-nucleotide polymorphisms [59,60].

#### 2.4.1. Physiologic ABCG2 Tissue Expression

Many studies have investigated the tissue distribution and the expression level of ABCG2, with the aim to decipher its physiologic function. High expression was found in the placenta, blood–brain barrier, prostate, mammalian glands, testis and ovaries, liver, small intestine, kidney, lung, and adrenal glands [61,62]. This strategic tissue localization suggests that ABCG2 plays a crucial role in limiting absorption, mediating distribution, and facilitating biliary and renal eliminations of drugs, and supporting the hypothesis of a protective role from dangerous xenobiotics [63].

##### Placenta

High ABCG2 expression has been found on the apical membrane of syncytiotrophoblasts of the chorionic villi, suggesting a protective role of the fetus from the possible transmission of toxins of maternal origin. In vivo experiments with intravenous administration of nitrofurantoin in *ABCG2*^−/−^ pregnant mice showed a five-fold higher fetal concentration compared to *ABCG2* wild-type mice [64]. An ex vivo placental vesicle system demonstrated a two-fold higher fetal concentration of the anti-diabetes drug glyburide when ABCG2 function was inhibited by novobiocin [65]. Moreover, Myllynen et al., demonstrated that a dietary carcinogen substrate of ABCG2 was transported against the concentration gradient from fetal to maternal circulation in a perfused placenta [66].

##### Blood–Brain Barrier (BBB)

Penetration of drugs through the BBB depends on the drug chemical properties but also on ABC protein activity, negatively affecting therapy efficacy in many neurological diseases, such as Alzheimer’s and Parkinson’s disease [67]. High ABCG2 expression was found in the luminal side of the micro-vessel endothelium of the brain [68,69]. It must be underlined that the penetration of any given substrate seems to be influenced by both ABCB1 and ABCG2, and the limitation of penetration could depend on the affinity of the substrate for each protein.

##### Mammary Gland

In contrast to what is described in the placenta, in the mammary gland, ABCG2 serves to concentrate toxins into milk. Many studies have demonstrated ABCG2-mediated high concentrations of antibiotics and anticancer drugs in milk [70,71]. Moreover, it seems to have a physiologic role in secreting vitamin B, required for the metabolism of the fat into milk [72].

##### Testis

High ABCG2 levels have been reported in interstitial cells as well in Sertoli/Leydig cells, confirming a protective role from genotoxic mutagens for the germinal stem cells [73]. High levels of ABCG2 have also been found in myoid cells and in luminal capillary endothelial cells, suggesting a barrier function like that of BBB [74].

##### Gastrointestinal Tract

Studies of expression revealed higher ABCG2 levels in the duodenum, then decreasing along the GI tract, with the lowest levels in the rectum. Protein distribution suggests a role in limiting the oral absorption of substrates [75]. This role was later confirmed by in vivo studies in ABCB1/ABCG2-deficient mice, where the availability of oral drugs was increased by 40% [76].

##### Kidney

ABCG2 is expressed in the cortical tubule and in the brush border membrane of the proximal tubule [77], suggesting a potential involvement of ABCG2 in renal drug excretion, a hypothesis supported by in vivo studies on *ABCG2^−/−^* mice compared to wild-type [78].

##### Liver and Biliary Tract

ABCG2 expression was found in the liver canalicular membrane, in hepatocytes, in the bile duct, and in the vascular endothelium of the human liver [73,79,80], having an active role in the biliary excretion of drugs, xenobiotics, and endogenous compound conjugates.

##### Hematopoietic Stem Cells

In a normal hematopoietic system, both murine and human, ABCG2 is expressed in a distinct population with a low proliferation rate and high bone marrow reconstitution capability after irradiation [81,82,83] and undergoes downmodulation during hematopoietic differentiation [84]. However, ABCG2 protein is not necessary for normal hematopoiesis, and *ABCG2*-deficient mice have normal peripheral cells; however, stem cells are more sensitive to mitoxantrone, an ABCG2 substrate [85].

### 2.5. ABCG2 Substrates

Since ABCG2 was first described, the list of its substrates has been steadily expanding. The substrate specificity of ABCG2 is highly overlapping to that of ABCB1, and like ABCB1, ABCG2 preferentially targets hydrophobic and lipophilic compounds with planar aromatic systems. Transfer across the membrane is associated with conformation changes of the protein (“in-facing”, with the substrate binding site open in the cytoplasm, and “out-facing” open in the extracellular space). Recently, Gyöngy et al., and Yu et al., proposed two molecular models to explain drug transport, highlighting the crucial role of ATP binding to modulate ABCG2 conformation [86,87]. As for the other ABC members, the molecular basis of ABCG2 substrate specificity is not fully elucidated. It has been hypothesized that substrate binding depends on the formation of a “membrane entrance” in the lipid bilayer by hydrophobic amino-acid residuals, available in the “in-facing” protein conformation, and that the different combination of these residuals provides the substrate specificity [88].

Mitoxantrone transport is the hallmark of the cells expressing ABCG2; thus, the first recognized substrates were predominantly chemotherapy agents. More recently, other classes of substrates have been identified, including antivirals, HMG CoA inhibitors, flavonoids, carcinogens, and calcium channel blockers [61,89]. A partial list of ABCG2 substrates is summarized in Table 1.

## 3. Expression and Clinical Significance of ABCG2 in AML

Despite the role of ABC proteins in determining drug resistance in hematologic and solid cancers, it is still a matter of controversy, and many studies in the past decades have shown a relationship between ABCG2 overexpression and poor clinical outcome in AML. The heterogeneity of the employed methods, including ABCG2 m-RNA expression, protein evaluation by flow cytometry or immune-cytochemistry, ABCG2 efflux of fluorescent substrates, and the lack of standardization may, in part, explain the confounding results and make data comparison difficult.

In 2001, Sargent et al., evaluated ABCG2 expression by immunocytochemistry, using the anti ABCG2 BXP-34 monoclonal antibody, in 20 samples of de novo AML (12 previously treated and 8 untreated). ABCG2 positivity showed high variability, but 27% patients had more than 10% positive cells and were considered as ABCG2 overexpressing. There were no differences between pretreated and naïve patients with regard to FAB cytotype or other clinical/biological characteristics. The authors also compared in vitro drug sensitivity in ABCG2 positive (>10%) and negative samples, observing significantly higher daunorubicin IC50 in ABCG2 positive cases [90].

Van del Kolk et al., tested ABCG2 expression and mitoxantrone efflux in 20 AML patients who were candidates for intensive chemotherapy. In all samples, ABCG2 expression was lower compared to the MCF7MR cell line, but a significant negative correlation between ABCG2 expression and mitoxantrone retention was demonstrated. Moreover, a higher expression in leukemic cells with more immature immunophenotypes (CD34+/CD38- and CD34+/CD33-) was observed, and the same subgroups also showed a reduced mitoxantrone retention compared to more mature leukemia cells, confirming the efflux activity of ABCG2. No ABCG2 upregulation was observed in refractory or relapsed patients [91].

Abbot et al., studied ABCG2 mRNA levels in 40 specimens from newly diagnosed adult AML patients. Only 7% showed ABCG2 mRNA levels within the range of drug resistant clones, although in another 78%, levels were higher than in normal blood and bone marrow. On this basis, they concluded that ABCG2 overexpression is uncommon in clinical specimens. Higher expression was reported in M0-M2 FAB cytotypes, while no correlation was found between ABCG2 mRNA level and cytogenetic or disease status (diagnosis vs. relapse) [92].

Van den Heuvel-Eibrink et al., compared ABCB1, ABCC1, LRP, and ABCG2 mRNA levels in 20 leukemia samples at diagnosis or relapse, observing significantly higher ABCG2 mRNA levels at relapse (median 1.7-fold, *p* = 0.04). On the contrary, expression levels of the other tested ABC proteins did not change. They hypothesized that only ABCG2 accounted for drug resistance at relapse [93]. In 2007, the same group explored the relevance of the same genes’ (ABCB1, ABCC1, LRP, and ABCG2) expression in a cohort of 154 elderly patients, observing a negative correlation between ABCB1 and ABCG2 expression and WBC count (*p* = 0.001) and a positive association between ABCB1 and ABCG2 and CD34 blast expression (*p* = 0.001). Moreover, high ABCB1/ABCG2 expression significantly reduced complete remission (CR) rate (*p* = 0.03) and seemed to be associated with a reduced event-free survival (EFS) (*p* = 0.05) [94].

Liu et al., reported a negative impact of ABC protein co-expression on prognosis. CR rate declined with increasing number of co-expressed ABC transporters; remission was 0% in cases with four-protein expression, 10% in those with three proteins, 25% in cases co-expressing two proteins, 58% in patients positive for only one protein, and 90% in negative patients (*p* = 0.001). Moreover, they reported significantly higher ABCG2 mRNA levels in patients older than 60 years (*p* = 0.008), in those with unfavorable cytogenetics (*p* = 0.017), and with FLT3-ITD or c-Kit mutation (*p* = 0.007 and *p* = 0.04, respectively) [95].

Marzac et al., analyzed the expression and the prognostic value of 22 ABC transporters in a cohort of 281 adult patients with AML, concluding that only ACBB1, ABCC1, and ABCG2 correlated with chemoresistance and had a negative impact on outcome [96].

Van der Pol et al., evaluated efflux function of ABCG2 in 26 leukemia samples. At diagnosis, 23/26 (88.5%) did not show efflux activity, while ABCB1 and ABCC1 activity was present in 36/45 (80%) and in 26/44 (59%) cases, respectively. No modifications were shown at AML relapse, so the authors concluded that ABCG2-mediated drug efflux offers limited contribution in developing resistance at relapse [97].

Galimberti et al., studied ABCG2 mRNA by real-time PCR in 52 AML samples, finding intermediate levels in 48.2% and high levels in 27.6%, and reporting a significant correlation between ABCB1 and ABCG2 values (r = 0.91, *p* = 0.0002). Neither ABCG2 nor ABCB1 levels correlated with clinical characteristics or cytogenetics, and no data on disease outcome was reported [98].

A negative impact of ABCG2 overexpression on disease outcome was reported by Benderra et al., in 149 adult AML patients, as high ABCG2 expression correlated with lower CR rate (*p* = 0.04) and OS (*p* = 0.05) compared to non-overexpressing patients. Furthermore, worse outcome was observed in patients co-expressing two or more ABC proteins [99].

Ho et al., investigated the expression of the entire ABC family to predict response to the classic “3 + 7” induction therapy in a small cohort of 34 AML patients. No association was demonstrated between ABC protein expression and response to therapy, but non-responding patients had higher levels of ABCB1 and ABCG2 in the candidate (i.e., CD34+/CD38-) leukemic stem cell population compared to responders [100]. The negative impact of ABCG2 expression on OS and disease-free survival (DFS) in adults was confirmed in the work of Uggla et al. [101].

Our group reported that the negative prognostic impact of ABCG2 overexpression cannot be reversed by adding fludarabine to induction therapy [102] nor by stem cell transplantation [103].

The prognostic significance of ABCG2 expression in pediatric AML was assessed by Steinbach et al., The authors evaluated ABCG2 expression by real time PCR in 59 pediatric cases, reporting 10-fold higher mRNA levels in patients not in remission after induction therapy compared to those achieving CR (*p* = 0.012). The highest expression was observed in the M1-M2 FAB subtype and the lowest in M5 (*p* = 0.004). Worse OS was reported in ABCG2 overexpressing cases, irrespective of disease risk (*p* = 0.023) [104]. The same group also investigated the prognostic relevance of the co-expression of ABCB1, ABCG2, ABCC3, and ABCA3 in 112 children with AML treated according to the AML-BFM 2004 protocol. Patients with high levels of ABCG2 and ABCC3 had reduced CR rate, and those with ABCG2 overexpression also had lower DFS. As for CR probability in adults (Liu et al. [95]), DFS in this pediatric cohort was negatively affected by the number of overexpressed ABC transporters (*p* < 0.001) [105].

Taken together, this data supports a role of ABCG2 in affecting AML outcome, both in adults and in children. Poor response to induction therapy may be also influenced by the frequent co-expression of ABCB1, but the stem cell–like properties conferred to leukemic cells by ABCG2 overexpression may account for high relapse rate and for poor survival when induction therapy is intensified or despite allogeneic stem cell transplantation, even if performed in CR.

## 4. ABCG2 Polymorphisms in AML

The cloning of ABCG2 DNA from drug-selected cell lines and from normal tissues revealed many amino-acid substitutions able to alter the protein function and the substrate preference. Moreover, mutational analysis identified more than 80 ethnic-associated synonymous and non-synonymous single-nucleotide polymorphisms (SNPs) potentially influencing ABCG2 expression and function and affecting drug absorption, plasma concentration, and distribution and elimination (Figure 1) [106].

In drug-selected cell lines such as S1-M1-80 and MCF7/AdVp3000, unique mutations in amino acid position 482 makes cells highly resistant to mitoxantrone and doxorubicin. The replacement of Arg with Gly or Thr at position 482 increases rhodamine and anthracycline efflux compared to the wild-type counterpart [107]. In contrast, the R482G and R482T variants negatively affect ABCG2’s ability to transport methotrexate but confer increased methotrexate resistance [108]. At least 13 ABCG2 variants with substitution at R482 have been described in cell lines, all associated with strong resistance to mitoxantrone. The COO- terminus of TMD3, near position 482, and 3D homology models suggest that R482 is the central cavity of the binding pocket, with a crucial function in drug transmembrane translocation [109]. Mutations at N557 and H630 confer lower resistance to SN-38, although mitoxantrone resistance is maintained [110]. Mutations at C603 may impair the homodimer formation [60], and substitutions at N596 may affect N-linked glycosylation, reducing the amount of ABCG2 on the cell membrane [111]. Among synonymous SNPs, Q141K has been associated with increased risk of gout in Asian [112] and American populations [113]. Moreover, Q141K SNP is associated with poor response to allopurinol [114]. In addition to Q141K, a Q126X SNP has been recognized as a risk factor for gout, and V12M SNP seems to have a protective effect in the Han Chinese population [115]. In a study of 229 Hungarian patients with late-onset Alzheimer’s Disease (AD) compared to 259 elderly non-dementia controls, the genotype C/C of 421C>A SNP (Q141K) was associated with increased susceptibility to AD (*p* = 0.024) [116]. Many ABCG2 SNPs have been recognized to affect anticancer drug transport and disposition. In AML, the two most common ABCG2 SNPs are rs2231137 and rs2231142, and the minor alleles of these SNPs are associated with a reduced level of ABCG2 expression [117]. The presence of rs2231137, rs2231142, and rs769188 variants in a cohort of 70 de novo adult AML patients did not influence anthracycline pharmacokinetics [118]. Studies on the impact of the ABCG2 polymorphism in response to therapy or toxicity in AML often report contradictory results. Data from the published studies are summarized in Table 2.

## 5. ABCG2 and Extracellular Vesicles

Among the factors mediating the acquisition of the MDR phenotype, the role of intercellular communications has recently emerged. In this setting, extracellular vesicles (EVs) play a pivotal role [124]. EVs are a heterogenous group of lipid bilayer structures derived from either endosomal multivesicular bodies (exosomes) or from the plasma membrane (micro vesicles, also called ectosomes). Cells that secrete more vesicles show a greater level of resistance [125]. Many studies have shown that cytotoxic drugs may be sequestered into EVs and released from the cells, thus preventing their accumulation in the nucleus. This mechanism has been demonstrated in MCF7 breast cancer–resistant cells, in which ABCG2 efflux protein localizes to EVs and mediates the uptake of drugs in the vesicles before their release [126]. The compartmentalization of ABCG2 in EVs and not in other intracellular compartments depends on the activation of the PI3K/AKT pathway, suggesting a potential therapeutic target to overcome drug resistance by the inhibition of EVs biogenesis. Preliminary in vitro studies using the specific PI3K-AKT axis results in the reduction of EV number and volume. ABCG2 is relocated to the intracellular compartment and loses the ability to concentrate anticancer drugs, thus restoring cell sensitivity [126]. EVs may act also by transferring between cells the efflux transporters or mi-RNAs involved in the regulation of efflux proteins’ expression [127]. Goler-Baron et al., recently proposed the use of photodynamic therapy in cancers and non-malignant diseases to destroy ABCG2-containing EVs previously treated with an ABCG2 substrate photosensitizer [128]. In the specific case of ABCG2 recipients, cells could develop resistance not only by increasing their capacity for extrude anticancer drugs but also by acquiring the protective characteristics typical of the stem cell compartment, mediated by ABCG2.

## 6. ABCG2 Inhibition

Different approaches to overcome ABCG2-mediated MDR have been proposed. Despite some positive results obtained in preventing ABCB1 binding [129,130], the attempt to develop chemotherapy agents that are not recognized by ABCG2 remains challenging, due to the molecular variety of transported compounds and the still incomplete knowledge of binding mechanisms [131]. The attractive strategy to induce collateral sensitivity (CS), a well-known phenomenon in which, due to overexpression of ABC transporters, a MDR cell becomes hypersensitive to some unrelated anticancer drugs, is difficult to pursue due to the variety of mechanisms involved in drug resistance [132].

So far, the most used method remains an inhibition of the efflux function, re-sensitizing resistant cells to conventional anticancer drugs. The first identified functional inhibitor of ABCG2 was fumitremorgin C (FTC), a mycotoxin produced by *Aspergillus fumigatus*, extensively used in experimental settings to test drug sensitivity in ABCG2 overexpressing cells [133]. Since then, using cellular approaches or in silico models, more than a hundred compounds in 45 different classes have been identified [134]. Some of them bind ABCG2 TMD, while others inhibit ATPase activity. Moinul et al., investigated many molecular scaffolds and relationships between structure and inhibition, proposing the “minimum” structural features required for ABCG2 inhibition, a potentially useful tool to design successful inhibitors [135].

It must be underlined that many of the molecules with inhibitory activity work only at high concentrations, often not attainable in clinical use. Among novel molecules, Ko143 (an FTC derivative), chromone derivatives, and a recently discovered indenoindol derivative are the most promising agents, having an IC50 in the nanomolar range [136]. Many ABCG2 inhibitors, like calcium channel blockers, anti-HIV drugs, and xanthine-oxidase inhibitors, were selected by drug repurposing, with the potential advantage of shortening the drug development process. The most interesting are tivozanib, fostamatinib, ponatinib, and febuxostat, all active at nanomolar IC50 [137,138,139,140]. A selected list of molecules that demonstrated in vitro inhibition of ABCG2-mediated efflux is reported in Table 3. The table incudes, for each compound class, only the molecules with the lowest IC50, thus potentially reachable in clinical use.

It must be underlined that, in leukemic cells, ABCG2 expression is significantly lower compared to cell lines and that an efficient inhibition could be obtained even at lower concentrations. Moreover, moving from the original molecule, modifications done according to critical requisites for ABCG2 inhibition could increase the potency of ABCG2 inhibitors while maintaining the bound affinity, to avoid off-target toxicities. Many inhibitors, supposedly selective for ABCG2, actually act as dual inhibitors, either anti-ABCG2/ABCB1 [142,143,144,145] or anti-ABCG2/ABCC1 [146]. Conversely, despite the overlap of substrates among the three ABC proteins involved in MDR, pan-inhibitors are rare. One is curcumin, which in vitro inhibits ABCB1, ABCC1, and ABCG2 [147]. A double/triple inhibition capacity may be useful since co-expression of ABC proteins is frequently observed in acute leukemias. A potential disadvantage could be a greater toxicity in tissues with physiological overexpression of ABC proteins.

Other new anticancer drugs have recently demonstrated an ABCG2 inhibitory effect in vitro. Sorf et al., demonstrated that ribociclib, a C4C/6 inhibitor approved for the treatment of locally advanced/metastatic breast cancer, inhibits ABCB1- and ABCG2-mediated daunorubicin and mitoxantrone efflux in AML cell lines at IC50 ranging between 1,4–3 μM, suggesting that combination therapy can revert MDR, especially in CD34+/FLT3-WT cases [148]. In vitro inhibition of ABCG2- and ABCC1-mediated daunorubicin and mitoxantrone efflux was demonstrated also by talazoparib, a drug approved for metastatic BCRA1/2 mutated breast cancer [149]. Finally, venetoclax, a bcl-2 inhibitor used in lymphoproliferative disease and in elderly AML patients in combination with hypomethylating agents, seems to inhibit the efflux function in wild-type ABCG2 in cell lines [150].

Despite the discovery of several molecules with an inhibitory effect, the interest in the clinical development of effective ABCG2 inhibitors has been deprioritized, likely due to the disappointing results attained in ABCB1 inhibition, and at present no clinical trials including ABCG2 inhibitors are ongoing. This is in contrast with the evident contribution of ABC transporters in chemotherapy failure, at least in AML. In this setting, ABCG2 overexpression not only accounts for an increased relapse risk and poor survival after conventional therapy, but also identifies a subset of patients at higher risk of relapse after allogeneic transplantation, which is still recognized as the only “curative” option for high-risk disease [103]. It is possible that the acquisition by ABCG2-overexpressing leukemic cells of stem-cell-like properties eventually favors their survival in transplant preparative regimens and their escape from post-transplant graft versus the leukemia effect. On this basis, strategies to counteract ABCG2 should be adopted.

Kukal et al., provided an exhaustive review of the network of signaling pathways involved in ABCG2 regulation [151]. This work highlights that most mechanisms affecting ABCG2 expression are also involved in leukemia pathogenesis. Drugs targeted against many of these mechanisms are already available or under trial. In vitro studies on cancer cell lines seem to confirm their ability to downregulate ABCG2 at transcription or post-translational level [134]. A selection of ABCG2 expression molecules according to regulation level is listed in Table 4.

With this basis, new chemotherapy protocols could be designed by combining conventional drugs and ABCG2 expression modulators, with the attempt to reverse stem cell properties in overexpressing AML blasts.

## 7. Conclusions

Great progress has been made in the past decades in deciphering ABCG2 structure, substrate transport, expression, and regulation as well as its impact on AML outcome. It is time to translate this knowledge into the clinics. New approaches should be developed to revert the negative effect of ABCG2 overexpression, including strategies addressed to down-modulate ABCG2 membrane expression, not only impair its efflux activity. The right inhibitor dose, the more appropriate administration timing (during induction therapy or after), the right chemotherapy association (conventional or hypomethylating agents to combine transcriptional and post-translational modulation), and the management of off-target toxicity and of the frequent co-expression of two or more ABC proteins remain open questions. Moreover, standardized methods for a precise “quantification” of ABCG2 expression should be established, based on protein rather than on mRNA measurement, due to the heavy post-transcriptional regulation of the protein.

## Figures and Tables

**Table 1:** Selected substrates of ABCG2 ^1^.

Chemotherapy Drugs
Daunorubicin, Doxorubicin, Idarubicin, Epirubicin, Etoposide, Gefitinib, Imatinib, Irinotecan, Mitoxantrone, Methotrexate, SN-38, Teniposide, Topotecan
Non-chemotherapy agents
Antibiotics: Ciprofloxacin, Ofloxacin, Norfloxacin, Erythromycin, Nitrofurantoin
Antivirals: Zidovudine, Lamivudine, Delavirdine, Lopinavir
Antihypertensive: Reserpine
Calcium channel blockers: Nicardipine, Azidopine, Nitrendipene, Dipyridamole
HMG-CoA reductase inhibitors: Rosuvastin, Cerivastatin, Pravastatin
Carcinogens: Aflatoxin B, 2-amino-1 methyl-6-phenyl-[4,5-b]imidazolpyridine (PhIP), 2-amino-3,8-dimethylimnidazo [4,5-f]quinoxaline (MelQx), 2-amini-3-methylimidazol[4,5-f]quinoline (IQ), 3-amini-1,4-dimethyl-5H-pyridol[4,3-b]indole (Trp-P-1) Others: Sulfasalazine, Cimetidine, Riboflavin, Vitamin K3, Glyburide, d-Luciferin, Quercetin

^1^ Table was compiled from references [61,62,63,64,65,66,67,68,69,70,71,72,73,74,75,76,77,78,79,80,81,82,83,84,85].

**Table 2:** Results of clinical studies for *ABCG2* polymorphisms in AML.

SNP	Author (Refs)	Disease Status	n	Ethnicity	Age (Range)	Chemotherapy	Outcome
G34A rs2231137	Hampras, 2010 [119]	De novo (75%) Secondary (25%)	261	Caucasian (86%) Others (14%) USA	61.5 (20–85)	ANT + AraC	- OS: GG↓OS (*p* = 0.05) (SCT censored) - Toxicity: AA/AG↑risk of toxicity grade ≥ 3
	Wang, 2011 [120]	De novo + ALL	141	Asian	32 (5–70)	AraC/Dauno/mitox	- CR: trend to↑CR (*p* = 0.053) - OS: GG↑OS (*p* < 0.001) - Haplotype GG (rs2231137) with CA(rs2231142) and CT (rs22331149), ↓DFS, OS (*p* < 0.001)
	Megías-Vericat, 2017 [121]	De novo	225	Caucasian	52.5 (16–78)	AraC/Ida	- CR, DDI: no influence - Toxicity: no influence
C421A rs2231142	Müller, 2008 [122]	De novo	139	Jews (61.2%) Arabs (38.8%)	46.3 (15–86)	AraC/Ant ± Fluda ± Mit	OS (SCT censored): no influence
	Hampras, 2010 [119,122]	De novo (75%) Secondary (25%)	261	Caucasian (86%) Others (14%) USA	61.5 (20–85)	ANT + AraC	- OS: no influence (SCT censored); Unadjusted HR: AA↓OS - Toxicity: no influence
	Wang, 2011 [120]	De novo + ALL	141	Asian	32 (5–70)	AraC/Dauno/Mit	- CR: no influence - OS:CC↑OS (*p* < 0.05, only univariate analysis). DFS: no influence * - Haplotype GG (rs2231137) with CA(rs2231142) and CT (rs22331149)↓DFS, OS (*p* < 0.001)
	Tiribelli, 2013 [123]	De novo	125	Caucasian (Italy)	59.2 (20–84)	AraC/IDA/Fluda ± Etop	- 3yOS:CC + low ABCG2↑OS (*p* = 0.02) - 3yDFS: CC + low ABCG2↑DFS (*p* = 0.04)
	Megías-Vericat, 2017 [121]	De novo	225	Caucasian	52.5 (16–78)	AraC/Ida	- CR, DDI: no influence - Toxicity: CA↑cardiac (*p* = 0.004),↑lung (*p* = 0.038)
Ile619Ile (C>T)	Wang, 2011 [120]	De novo + ALL	141	Asian	32 (5–70)	AraC/Dauno/mitox	- CR, OS, DFS: no influence *
rs2231149 (C>T)	Wang, 2011 [120]	De novo + ALL	141	Asian	32 (5–70)	AraC/Dauno/mitox	- CR: no influence - OS: CC↑OS (*p* = 0.01; lost in multivariate analysis) - DFS: CC↑DSF (*p* < 0.05; lost in multivariate analysis) * - Haplotype GG (rs2231137) with CA(rs2231142) and CT (rs22331149), ↓DFS, OS (*p* < 0.001)
rs2231162	Wang, 2011 [120]	De novo + ALL	141	Asian	32 (5–70)	AraC/Dauno/mitox	CR, OS, DFS: no influence *
rs2231164	Wang, 2011 [120]	De novo + ALL	141	Asian	32 (5–70)	AraC/Dauno/mitox	CR, OS, DFS: no influence *
ABCG2 + SLC							
ABCG2 rs2231142(C>A) SLC22A16 rs714368(A>C)	Megías-Vericat, 2017 [121]	De novo	225	Caucasian	52.5 (16–78)	AraC/Ida	- CR, DDI: no influence - Toxicity:genotype AC + AA:↑cardiac (*p* = 0.033)

* Mixed with ALL cases. Abbreviations: AML: acute myeloid leukemia; ALL: acute lymphoblastic leukemia; ANT: anthracycline; Dauno: daunorubicin; IDA: idarubicin; Mit: mitoxantrone; Fluda: fludarabine; Etop: etoposide; CR: complete remission; DFS: disease-free survival; OS: overall survival; SCT: stem cell transplantation; HR: hazard risk. Arrow up = increased; arrow down = decreased/reduced.

**Table 3:** Selected list of ABCG2 inhibitors by class of compounds ^1^.

Structural Class Title 1	Compound	IC50 (μM)
Chalcones	Indolylphenylproenone	0.27
Chromones	Chromone4a	0.086
	Chromone31	0.046
Diketopiperazines	Ko143 (FTC analog)	0.01
Flavonois	Flavone	2.8
	6-prenulchrysin	0.29
	Flavonoid dimer	1
Hedgehog pathway inhibitors	Vismodegib	1.4
Immunosuppressants	Sirolimus	1.9
Non-purine xanthine oxidase inhibitors	Febuxostat	0.027
	Topiroxostat	0.18
ABCB1 inhibitors	Tariquidar	0.9
	Tariquidar derivative 6	0.06
Indenoindole-type derivatives	Indeno[1,2-b]indole	0.21
	9-hydroxyindeno[1,2-b]indole	0.21
	Indeno[1,2-b]indole homodimer	0.024
Tariquidar-related triazoles	IR-MB19	0.14
	UR-MB108	0.079
Thrombopoietin receptor	Eltrombopag	3.1
Tyrosine kinase inhibitors	Alectinib	1.5
	Bosutinib	2
	Dasatinib	2
	Erlotinib	0.13
	Fostamatinib	0.05
	Gefitinb	0.5
	Ponatinib	0.04
	Vandetanib	0.2
	Tivozanib	0.07
	Imatinib	1

^1^ Only molecules with low IC 50 are included. Inhibitory activity was tested in stabilized cancer cell lines of different origin. The list was compiled from references [134,141].

**Table 4:** ABCG2 expression modulators according to regulation level.

Transcriptional Regulation	Post-Translational Regulation
Dexamethasone Genistein Resveratrol Gefitinib LY294002 (PI3K inhibitor) Glasdegib Vadadustat SP600125 (JNK inhibitor) Telatinib	Imatinib Nilotinib Dasatinib Sorafenib Gefitinib LY294002 (PI3K inhibitor) PPAR-γ agonists (telmisartan)

List was compiled from references [89,134].

## Data Availability

Not applicable.
